# Microcephaly family protein MCPH1 stabilizes RAD51 filaments

**DOI:** 10.1093/nar/gkaa636

**Published:** 2020-07-31

**Authors:** Hao-Yen Chang, Chia-Yi Lee, Chih-Hao Lu, Wei Lee, Han-Lin Yang, Hsin-Yi Yeh, Hung-Wen Li, Peter Chi

**Affiliations:** Institute of Biochemical Sciences, National Taiwan University, No. 1, Sec. 4, Roosevelt Road, Taipei 10617, Taiwan; Institute of Biochemical Sciences, National Taiwan University, No. 1, Sec. 4, Roosevelt Road, Taipei 10617, Taiwan; Department of Chemistry, National Taiwan University, No. 1, Sec. 4, Roosevelt Road, Taipei 10617, Taiwan; Department of Chemistry, National Taiwan University, No. 1, Sec. 4, Roosevelt Road, Taipei 10617, Taiwan; Department of Chemistry, National Taiwan University, No. 1, Sec. 4, Roosevelt Road, Taipei 10617, Taiwan; Institute of Biochemical Sciences, National Taiwan University, No. 1, Sec. 4, Roosevelt Road, Taipei 10617, Taiwan; Department of Chemistry, National Taiwan University, No. 1, Sec. 4, Roosevelt Road, Taipei 10617, Taiwan; Institute of Biochemical Sciences, National Taiwan University, No. 1, Sec. 4, Roosevelt Road, Taipei 10617, Taiwan; Institute of Biological Chemistry, Academia Sinica, 128 Academia Road, Section 2, Nankang, Taipei 11529, Taiwan

## Abstract

Microcephalin 1 (MCPH1) was identified from genetic mutations in patients with primary autosomal recessive microcephaly. In response to DNA double-strand breaks (DSBs), MCPH1 forms damage-induced foci and recruits BRCA2–RAD51 complex, a key component of the DSB repair machinery for homologous recombination (HR), to damage sites. Accordingly, the efficiency of HR is significantly attenuated upon depletion of MCPH1. The biochemical characteristics of MCPH1 and its functional interaction with the HR machinery had remained unclear due to lack of highly purified MCPH1 recombinant protein for functional study. Here, we established a mammalian expression system to express and purify MCPH1 protein. We show that MCPH1 is a *bona fide* DNA-binding protein and provide direct biochemical analysis of this MCPH family protein. Furthermore, we reveal that MCPH1 directly interacts with RAD51 at multiple contact points, providing evidence for how MCPH1 physically engages with the HR machinery. Importantly, we demonstrate that MCPH1 enhances the stability of RAD51 on single-strand DNA, a prerequisite step for RAD51-mediated recombination. Single-molecule tethered particle motion analysis showed a ∼2-fold increase in the lifetime of RAD51–ssDNA filaments in the presence of MCPH1. Thus, our study demonstrates direct crosstalk between microcephaly protein MCPH1 and the recombination component RAD51 for DSB repair.

## INTRODUCTION

Autosomal recessive primary microcephaly in human, also termed MicroCephaly Primary Hereditary (MCPH), is characterized by a reduced head size due to abnormal neurodevelopment (1). Apart from microcephaly, patients also suffer from developmental delay, mental retardation, and infertility (2). Recent studies have further documented that Microcephalin 1 (*MCPH1*) dysfunction is highly correlated with tumorigenesis and prognostic survival (3–6), indicative of a tumor suppressor function.

MCPH1 conditional knockout mice exhibit growth retardation, infertility and reduced brain size (7–10), recapitulating phenotypes of human patients. Dysfunctional meiotic recombination in spermatocytes has been attributed as the cause of the infertility phenotype (7). Relative to wild-type mice, fetal neurospheres in *Mcph1*^−/−^ mice were not affected, but numbers of infant neurospheres were reduced, with this phenomenon correlating with defective proliferation of neuroprogenitor cells (8,9). Two possible mechanisms may account for this loss of neuroprogenitor cells. Firstly, *Mcph1*^−/−^ cells were observed to undergo premature chromosome condensation (PCC), uncoupled mitosis and centrosomal cycling, resulting in disorientated mitotic spindle and thereby altering the division plane and increasing the asymmetric divisions that perturb neurogenic cell fate (8). Secondly, *Mcph1*^−/−^ neural cells exhibited sensitivity to DNA damage, resulting in an accumulation of apoptotic cells (8,9). Consistent with this latter notion, double-strand break (DSB) repair pathways, including homologous recombination (HR) and non-homologous end-joining (NHEJ), are compromised in *Mcph1^−^^/^^−^* mouse embryonic fibroblasts (MEF) (9,10). However, how MCPH1 facilitates DSB repair remains unclear. One potential explanation is that MCPH1 plays a proximal role in regulating the ATM and ATR pathways to manage downstream DSB repair (3,11–13). In response to DNA damage, MCPH1 forms damage-induced foci and cooperates with γ-H2AX to recruit DNA damage response signaling components including ATM, NBS1, and MDC1 (3,14–18). Notably, MCPH1 interacts with SWI/SNF complex to facilitate chromatin relaxation and to recruit DNA repair proteins (13). However, if and how MCPH1 directly regulates the DNA repair machinery has remained an open question.

HR-mediated DSB repair is indispensable for the maintenance of genomic integrity (19–25). During HR, the assembly of RAD51 nucleoprotein filament onto single-strand DNA (ssDNA) derived from nucleolytic processing of a primary DNA lesion is a prerequisite step for the homologous duplex template searching and DNA strand exchange necessary to fix strand breaks (19–22). BRCA2 plays an essential role in facilitating RAD51 filament assembly by competing with the high-affinity ssDNA-binding protein RPA for ssDNA substrates (26). It has been well documented that depletion of MCPH1 causes impaired loading of both BRCA2 and RAD51 onto DNA damage sites, thereby attenuating recombination efficiency (7,9,10,27,28). Immunoprecipitation of MCPH1 with both RAD51 and BRCA2 suggests that a functional interaction exists between MCPH1 and the HR machinery (7,27). In addition to its function in mitotic recombination, the infertility induced by MCPH1 deficiency indicates an extended role of MCPH1 in meiotic recombination. In mouse *Mcph1*-deficient spermatocytes, meiosis was arrested prior to the pachytene stage and displayed aberrant chromosomal synapsis. Localization of RAD51 or BRCA2 at meiotic chromosomes was also impaired, and γ-H2AX accumulation revealed incomplete DNA crossover (7). However, the functional role of MCPH1 in RAD51-mediated recombination remains largely uncharacterized due to a lack of purified MCPH1 recombinant protein for biochemical study. Here, we present an efficient protocol for expressing and purifying MCPH1 in native form. Remarkably, our biochemical and single-molecule analyses reveal that purified MCPH1 protein possesses DNA-binding activity and stabilizes RAD51 nucleoprotein filaments. Thus, our study reveals the biochemical characteristics of MCPH1 and how this MCPH family member directly regulates RAD51 filament stability.

## MATERIALS AND METHODS

### DNA substrates

The Φx174 viral (+) strand ssDNA and replicative form I dsDNA were purchased from New England BioLabs. Oligonucleotides were gel-purified as previously described (29). To prepare 5′-end-labeled ssDNA for Benzonase protection assays, 80-mer Oligo 1 was incubated with polynucleotide kinase (New England Biolabs) and [γ-P^32^-ATP] (PerkinElmer) for 5′-end-labeling. The unincorporated nucleotide was removed using a Spin 6 column (Bio-Rad). For oligonucleotide-based DNA binding, the DNA substrates Oligos 2–4 and Oligos 5–10 were derived from previous researches (30,31). The sequences of DNA substrates used in our DNA mobility shift and Benzonase protection assays are summarized in Supplementary Table S1, and the composition of annealed DNA substrates for oligonucleotide-based DNA binding is summarized in Supplementary Table S2. For tethering particle motion analysis, a gapped DNA substrate (151 bp dsDNA handle + (dT)135 nt ssDNA + 19 bp dsDNA handle) was prepared by annealing the PCR products as described in previous reports (32,33).

### Plasmids

**Figure 1.**
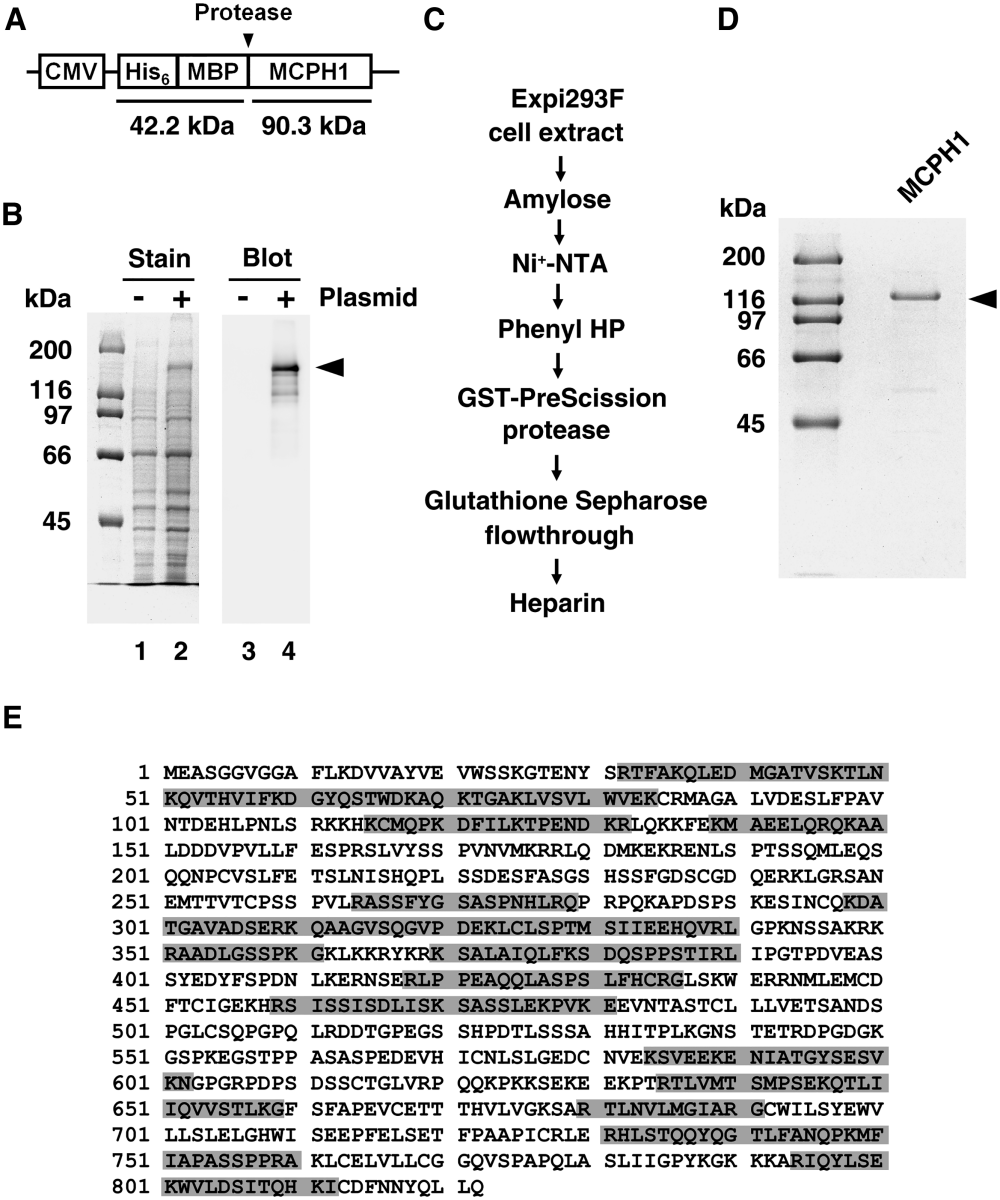
Expression, purification, and identification of MCPH1 protein. (**A**) The expression plasmid of MCPH1. (**B**) The MCPH1 expression plasmid was transfected and expressed in human Expi293F cells. Whole cell extract was analyzed in a 10% SDS-denaturing polyacrylamide gel and stained with Coomassie Blue (stain) or western blot with anti-MCPH1 antibody (blot). (**C**) Schematic of our purification protocol. The Expi293F cell extract was fractionated through amylose, Ni^+^-NTA, phenyl HP and heparin columns. The MBP tag was removed using GST-tagged PreScission protease and then filtered through a GST column to remove the protease. (**D**) Purified MCPH1 protein (1 μg), as analyzed in a 12% SDS-denaturing polyacrylamide gel with Coomassie Blue staining. (**E**) Results from ESI-MS/MS analysis of purified MCPH1. Identified fragments are shaded in gray.

To obtain the wild-type MCPH1 expression plasmid, (His)_6_-tagged Maltose binding protein (MBP) cDNA, a cutting site for PreScission protease, and mouse MCPH1 cDNA (NM_173189.2) were ligated into the EcoRI and NotI sites of pcDNA3.4 (Thermo Fisher) using a HiFi DNA Assembly Kit (New England Biolabs). The (His)_6_-MBP tag was attached to the amino-terminus of the protein, as indicated in Figure 1A. For the expression plasmids of truncated MCPH1 variants in Supplementary Figure S4A, (His)_6_-SUMO and PCR-truncated mouse MCPH1 cDNAs were inserted into the NcoI and NotI sites of pRSFDuet (Novagen), enabling addition of a (His)_6_-SUMO tag to the amino-terminus of the proteins. All expression plasmids were sequenced to ensure that no unexpected mutations arose during the cloning process.

### Protein expression

To express mouse MCPH1, Expi293F cells (Thermo Fisher; maintained in Expi293 Expression Medium with an orbital shaker, 8% CO_2_, 37°C) were cultured to 2.0 × 10^6^ viable cells/ml prior to transfection. We transfected 1 μg plasmid DNA per ml of cells according to the instructions of the ExpiFectamine 293 Transfection kit (Thermo Fisher). After 48 h of incubation, protein expression was assessed upon cell lysis with 2% SDS and analyzed by means of 10% SDS-PAGE and western blotting with MCPH1 antibody (rabbit mAb #4120, Cell Signaling). For truncated MCPH1 variants, BL21*(DE3)(pLys) *Escherichia coli* strain harboring plasmids expressing amino-terminally-tagged (His)_6_-SUMO-MCPH1 truncated variants were cultured in Luria broth at 37°C to OD_600_ = 0.6-0.8, followed by addition of 1 mM isopropyl-1-thio-β-d-galactopyranoside for 3 h at 37°C to induce protein expression.

### Protein purification

All purification steps were carried out at 4°C. To purify (His)_6_-MBP-tagged mouse MCPH1, cell paste from 1.2 l culture was suspended in 100 ml of buffer A (25 mM Tris–HCl pH 7.5, 10% glycerol, 0.01% Igepal, 2 mM β-mercaptoethanol, 0.5 mM EDTA) containing 300 mM KCl, protease inhibitors (aprotinin, chymostatin, leupeptin and pepstatin A at 2 μg/ml each; 0.1 mM PMSF, and 1 mM benzamidine), and then subjected to sonication. The clarified lysate was centrifuged (100 000 × *g* for 1 h) and then incubated with 4 ml Amylose resin (New England Biolabs) for 4 h. The matrix was poured into a column and washed with 50 ml buffer A with 2 mM MgCl_2_ and 2 mM ATP to remove chaperone proteins. The tagged MCPH1 was eluted with 20 ml of buffer A containing 300 mM KCl and 20 mM Maltose. The eluate was then mixed with 3 ml Ni^+^-NTA beads for 8 h. The mixture was poured into a column and washed with 50 ml buffer A in addition to 300 mM KCl, 5 mM imidazole, 2 mM MgCl_2_ and 2 mM ATP to remove non-specific proteins completely. The tagged MCPH1 was eluted with 15 ml of buffer A containing 300 mM KCl and 200 mM imidazole. The tagged MCPH1-containing fractions were pooled and fractionated in a 1 ml HiTrap Phenyl-HP column (GE Healthcare) using a 20 ml gradient of 200–500 mM (NH_4_)_2_SO_4_ in buffer B (20 mM K_2_HPO_4_ pH 7.5, 10% glycerol, 50 mM KCl, 0.01% Igepal, 1 mM β-mercaptoethanol, and 0.5 mM EDTA). To obtain tag-free MCPH1, 0.3 mg (His)_6_-MBP-tagged MCPH1 protein from the Phenyl-HP elution was digested with 245 μg PreScission protease for 6 h, and then the mixture was poured through Glutathione Sepharose to remove GST-tagged protease. Next, the cleaved or un-cleaved MCPH1 fraction was fractionated in a 1 ml Heparin column (GE Healthcare) using a 6 ml gradient of 380–620 mM KCl in buffer A. The MCPH1-containing fractions were pooled and concentrated in an Ultracel-100K concentrator (Millipore). The concentrated preparation was divided into small aliquots, subjected to N_2_ freezing, and stored at −80°C. Aliquots were qualified by Bradford protein assay (Bio-Rad) and Coomassie Blue staining.

The (His)_6_-SUMO-tagged truncated MCPH1 variants were lysed in buffer A containing 300 mM KCl and protease inhibitors, and then subjected to sonication as described above. The clarified lysate was prepared after centrifugation (18 000 × *g* for 20 min), and then incubated with Ni^+^-NTA beads for 1 h. The beads were further washed with 1 ml buffer A to which was added 300 mM KCl and 5 mM imidazole. The truncated variants were eluted as per the Ni^+^-NTA step described above. The eluates were qualified by Coomassie Blue staining and stored at −80°C.

Strep-tagged or (His)_6_-tagged mouse RAD51 and mouse DMC1 proteins were purified as described previously (29,34). *Escherichia coli* RecA protein was purchased from New England Biolabs.

### Mass spectrometry analysis

Mass spectrometry (MS) analysis for protein identification was conducted by VISUAL PROTEIN (http://www.visualprotein.com/). To prepare MS samples, purified MCPH1 was resolved by SDS-PAGE and spots excised from the stained gels were processed according to a standard MS sample preparation protocol (35,36). Reduction was performed using 10 mM dithiotreitol and 25 mM Ammonium bicarbonate for 45 min at 56°C. Then, alkylation was achived using 5% 4-vinylpyridine, 25 mM ammonium bicarbonate and 50% acetonitrile for 30 min at room temperature in the dark. In-gel digestion of proteins was carried out overnight at 37°C using MS-grade Trypsin Gold (Promega, Madison, WI, USA). Trypsin-digested products were extracted initially using 10 μl Milli-Q water, followed by two sequential extractions with 50% acetonitrile and 0.1% trifluoroacetic acid (in a total volume of 20 μl). The combined extracts were dried in a vacuum concentrator at room temperature, and then dissolved in 1 μl of 5% acetonitrile and 0.5% Trifluoroacetic acid.

A Thermo LTQ-Orbitrap system (Thermo Scientific, UK) was used for ESI-MS/MS spectrometry. The MS/MS signal was analyzed using the MASCOT search engine (http://www.matrixscience.com). The search parameters were defined as follows: taxonomy, *Mus musculus*; enzyme, trypsin; fixed modification, pyridylethylation; variable modifications, oxidation; peptide MS tolerance, ±40 ppm; fragment MS tolerance, ±1 Da and allowance of one missed cleavage.

### DNA mobility shift assay

The Φx174 viral (+) strand ssDNA (6 μM nucleotides) or supercoiled Φx174 replicative form I dsDNA (6 μM base pairs) was incubated with the indicated amounts of MCPH1 protein in 10 μl of reaction buffer C (35 mM Tris–HCl pH 7.5, 1 mM DTT, 50 mM KCl, 0.1 μg/μl BSA) at 37°C for 5 min. The reaction mixtures were electrophoresed in 1% agarose gels in TBE buffer (89 mM Tris, 89 mM borate and 2 mM EDTA pH 8.0) at 4°C, and the DNA species were then stained with SYBR Gold (Thermo Fisher). The gels were analyzed in a gel documentation station (Bio-Rad).

For oligonucleotide-based DNA binding, oligo DNA substrates (750 nM nucleotides and base pairs) were incubated with the indicated amounts of MCPH1 proteins in 10 μl of reaction buffer C at 37°C for 5 min. The reaction mixtures were electrophoresed in 6% polyacrylamide gels in TBE buffer at 4°C. The gels were dried on DE81 paper and the DNA species were quantified by phosphorimaging analysis in a Personal FX phosphorimager using the Quantity One software (Bio-Rad). The bound percentage of MCPH1–DNA complex was calculated from the ratio of decreasing intensity of un-bound DNA to native DNA.

### Affinity pulldown

(His)_6_-MBP-tagged MCPH1 (4 μg) was incubated with either Strep-tagged mouse RAD51 (1 μg), Strep-tagged mouse DMC1 (1 μg), or tag-free *E. coli* RecA (1 μg, New England Biolabs) in 30 μl reaction buffer D (25 mM Tris–HCl pH 7.5, 10% glycerol, 0.01% Igepal, 100 mM KCl, 10 mM imidazole) for 30 min at 37°C. Benzonase (10 units) and MgCl_2_ (2 mM) were included in the reactions to remove any possible nucleic acid contamination. Upon mixing with 10 μl of Talon resin (Clontech) for 30 min at 25°C to capture the (His)_6_-tagged protein and associated recombinases, the resins were washed three times with 30 μl reaction buffer and then treated with 25 μl of 2% SDS at 55°C to elute the proteins. The supernatant, first wash, and SDS eluate (10 μl of each) were analyzed by 12% SDS–PAGE with Coomassie Blue staining. The same procedure was used to pull down (His)_6_-SUMO-tagged mouse MCPH1 fragments (2 μg) with Strep-tagged mouse RAD51 (2 μg) or Strep-tagged mouse DMC1 (2 μg), as depicted in Supplementary Figures S4 and S5. For comparison, the supernatant and SDS eluate (1 μl of each) were analyzed by means of 12% SDS-PAGE and western blotting with RAD51 antibody (rabbit pAb sc-8349, Santa Cruz) and DMC1 antibody (mouse mAb GTX11054, GeneTex). The intensity of individual protein bands was quantitated using Quantity One software (Bio-Rad). The percentage of recombinase binding was calculated from the ratio of eluted recombinase to total recombinase for both the supernatant and the eluate.

### Benzonase protection assay

We incubated (37°C for 5 min) 0.5 μM each of (His)_6_-tagged mouse RAD51, (His)_6_-tagged mouse DMC1 or (His)_6_-tagged *E. coli* RecA (New England Biolabs) with 5′-end ^32^P-labeled 80-mer Oligo 1 ssDNA (1.5 μM nucleotides) to assemble filament in 8 μl reaction buffer C to which was added 2 mM MgCl_2_ and 0.1 mM ATP. Then, indicated amounts of tag-free MCPH1 were included in the reaction mixtures for a further 5 min. To challenge filament stability, Benzonase (Endonuclease, 20 units; New England Biolabs) was added into the reaction mixtures (10 μl final volume). After 10 min of incubation at 37°C, the reaction mixtures were mixed with 2.5 μl of termination buffer (240 mM EDTA, 2% SDS and 3.2 mg/ml proteinase K) and incubated at 37°C for 10 min. The reaction mixtures were resolved in 10% polyacrylamide gels in TBE buffer at 4°C. The gels were dried on DE81 paper and the DNA species were quantified by phosphorimaging analysis in a Personal FX phosphorimager using the Quantity One software (Bio-Rad). The percentage of DNA protected was calculated as the ratio of the intensities of undigested DNA to total DNA, with the intensity of the DNA blank being defined as 100%.

### Tethered particle motion disassembly assay

The tethered particle motion disassembly experiment was performed as described previously (37) with minor modification. To prepare the siloxane surface, the glass slides were sequentially sonicated in 2 M KOH for 5 min, 99% ethanol for 10–15 min and ddH_2_O for 15–20 min. After these sonication steps, the slides were rinsed with ddH_2_O and dried with N_2_ gas. The glass slides were then functionalized in a solution containing 1,7-dichloro-octamethytetrasiloxane (Sigma-Aldrich) in 99% ethanol in the dark overnight at room temperature. The slides were then alternatively rinsed with 99% ethanol and ddH_2_O before drying with N_2_ gas. Gapped DNA substrates (151 bp dsDNA handle + (dT)135 nt ssDNA + 19 bp dsDNA handle) were anchored specifically on the slide by means of digoxigenin/anti-digoxigenin linkage and were attached to Streptavidin-coated beads. The DNA tethers were pre-incubated with mixtures of 0.8 μM (His)_6_-tagged mouse RAD51 in reaction buffer E (30 mM Tris–HCl pH 7.5, 1 mM DTT, 2 mM ATP, 2 mM MgCl_2_, 2 mg/ml BSA and 100 mM KCl) to form nucleoprotein filaments. After 5–10 min incubation, free RAD51 was removed by three washes with 30 μl reaction buffer containing ATP regeneration system (1 mM phosphoenolpyruvate and 4 units/ml pyruvate kinase). This extensive washing did not disrupt protein filaments remaining at the end of the wash. The time of the final wash was defined as time zero and the DNA tethers were monitored for ∼15–20 min thereafter to assess the disassembly process. For experiments involving MCPH1, pre-formed RAD51 nucleoprotein filament was washed twice as described above to remove free RAD51. On the third wash, RAD51 filament was subjected to reaction buffer containing ATP regeneration system and the indicated amounts of MCPH1 to form MCPH1-associated RAD51 nucleoprotein filament. Tethers with Brownian motion (BM) ranging from 35 to 80 nm were scored as RAD51 nucleoprotein filaments. For disassembly of DMC1 filaments, the working condition is followed as described above. The lifetime of RAD51 filament was determined as the time-point when BM continuously declined (37). The reported lifetime was obtained from cumulative survival histograms using the Kaplan–Meier estimator (38).

## RESULTS

### Expression and purification of mammalian MCPH1

To characterize the biochemical properties of microcephalin 1 protein, we used mouse MCPH1 cDNA to produce recombinant protein for two reasons. First, there is only one protein isoform in mouse rather than the eight isoforms in human cells. Second, MCPH1-null mice exhibit a similar pathology to that of human patients with autosomal recessive microcephaly, especially in terms of defective DNA repair (7–10). Accordingly, we constructed an expression vector harboring mouse MCPH1 with a maltose-binding protein (MBP) tag at its amino terminus to enhance protein solubility and stability in our mammalian cell expression system (Figure 1A). Recombinant MCPH1 protein was expressed and then identified by western blotting after 48 h of transfection (Figure 1B). The cell lysate was processed through a sophisticated purification procedure, as described in the MATERIAL AND METHODS, to obtain recombinant MCPH1 proteins with or without a tag (Figure 1C and Supplementary Figure S1A). The (His)_6_-MBP-tagged and tag-free MCPH1 exhibited purities ≥90% and no nuclease contamination impaired subsequent functional analyses (Figure 1D and Supplementary Figure S1B). Importantly, gel filtration analyses further show that purified MCPH1 is not a soluble aggregate (Supplementary Figure S2). The purified, untagged MCPH1 protein (calculated molecular mass of 90,372 Da) migrated in SDS-PAGE analysis as a species of ∼120 kDa (Figure 1D), and its identity was further confirmed by liquid chromatography–tandem mass spectrometry (LC–MS/MS) (Figure 1E). Note, tagged (His)_6_-MBP-MCPH1 proteins were used only in affinity pulldown experiments, whereas tag-free MCPH1 proteins were used in all biochemical and single-molecule TPM experiments. Consequently, we obtained a high purity of tag-free MCPH1 recombinant protein for the subsequent biochemical analyses described below.

### MCPH1 is a *bona fide* DNA-binding protein

**Figure 2.**
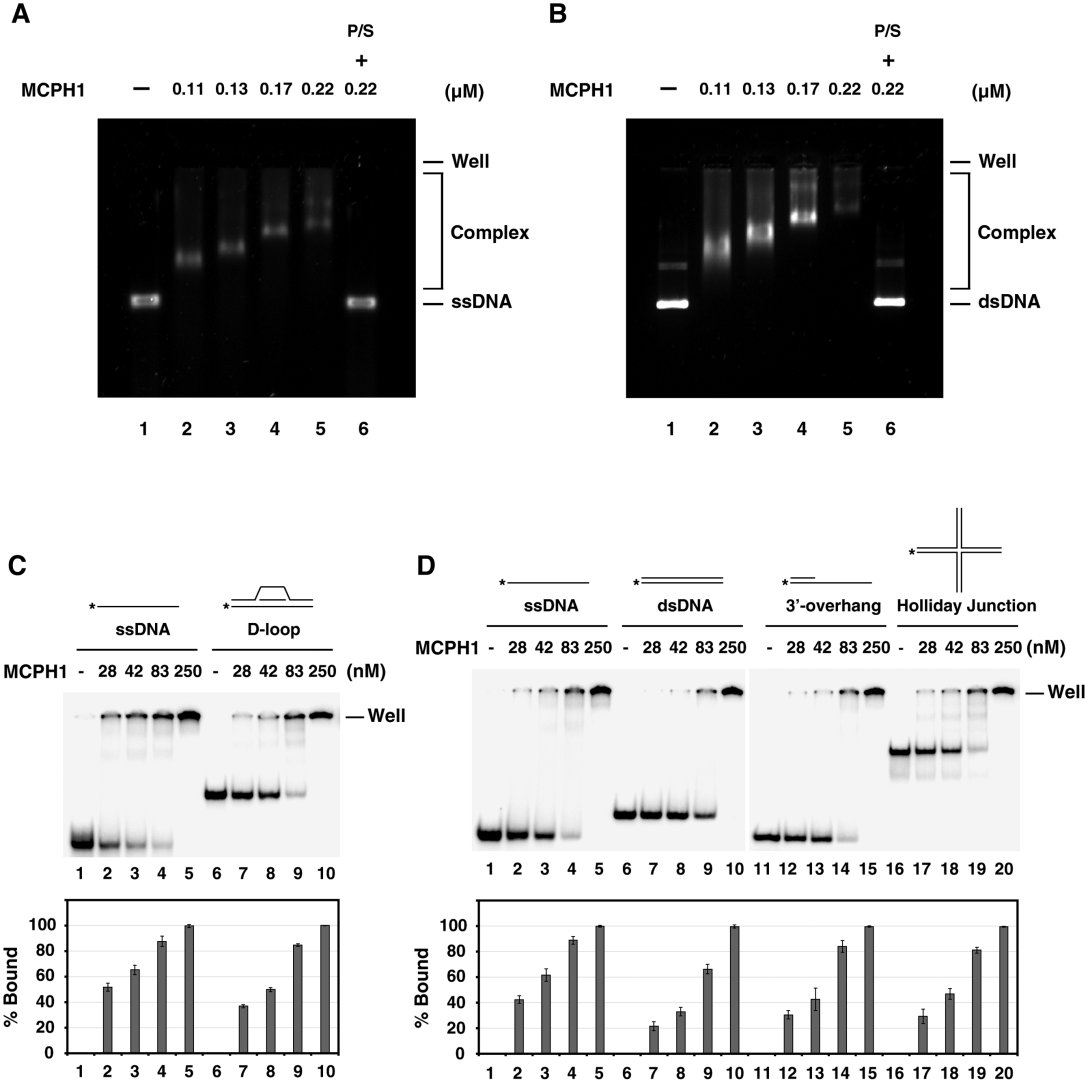
MCPH1 possesses DNA-binding activity. (**A**) Indicated amounts of tag-free MCPH1 were incubated with circular Φx174 ssDNA (6 μM nucleotides). (**B**) or supercoiled Φx174 dsDNA (6 μM base pairs). The DNA species were then revealed in 1% TBE agarose gels and stained with SYBR gold. In lane 6, the reaction mixture was treated with proteinase K and SDS (P/S) to release the DNA from the nucleoprotein complex. To assess oligo DNA binding, indicated amounts of MCPH1 were incubated with isotope-labeled DNA substrates (750 nM nucleotides or base pairs) as follows: (**C**) ssDNA (Oligo 3, lanes 1–5) or D-loop (Oligo 2 + 3 + 4, lanes 6–10) and (**D**) ssDNA (Oligo 5, lanes 1–5), dsDNA (Oligo 5 + 9, lanes 6–10), 3′-overhang DNA (Oligo 5 + 10, lanes 11–15) or Holliday junction (Oligo 5 + 6 + 7 + 8, lanes 16–20). The ^32^P-label is denoted by the asterisk. The DNA species were revealed in 6% polyacrylamide gels in TBE buffer, the gels were dried, and then analyzed by phosphorimaging. Percentage bound DNA is shown below the gels. Error bars represent the standard deviation (±SD) calculated from three independent experiments.

MCPH1 was previously shown to tightly associate with chromatin based on observations that MCPH1 forms DNA damage-induced foci and is enriched in chromatin fractions of cell lysates (7,27). These findings raise the important question as to whether MCPH1 possesses DNA-binding ability. To investigate this issue, we used an electrophoretic mobility shift assay (EMSA) to analyze the DNA-binding activity of MCPH1. MCPH1 exhibited dosage-dependent DNA-binding activity on both circular ΦX174 single-strand DNA (ssDNA) and supercoiled double-strand DNA (dsDNA) (Figures 2A and B). Binding efficiencies of MCPH1 to both these DNA substrates were similar. For comparison, we tested MCPH1 DNA-binding activity for short oligonucleotides of ssDNA, dsDNA, over-hang DNA, D-loop and Holliday junction (Supplementary Table S2). This analysis indicated that MCPH1 does not display a significant DNA-binding preference, though it does bind slightly better on ssDNA (Figure 2C and D). To confirm the DNA-binding ability of MCPH1, we raised the KCl content of our EMSA assay to 300 mM (i.e. above physiological relevant conditions) and found that ∼80% MCPH1-DNA complex formed relative to at a KCl content of 50 mM (Supplementary Figure S3). Thus, our *in vitro* biochemical analysis reveals that MCPH1 possesses DNA-binding activity.

### MCPH1 physically interacts with both RAD51 and DMC1 recombinases

**Figure 3.**
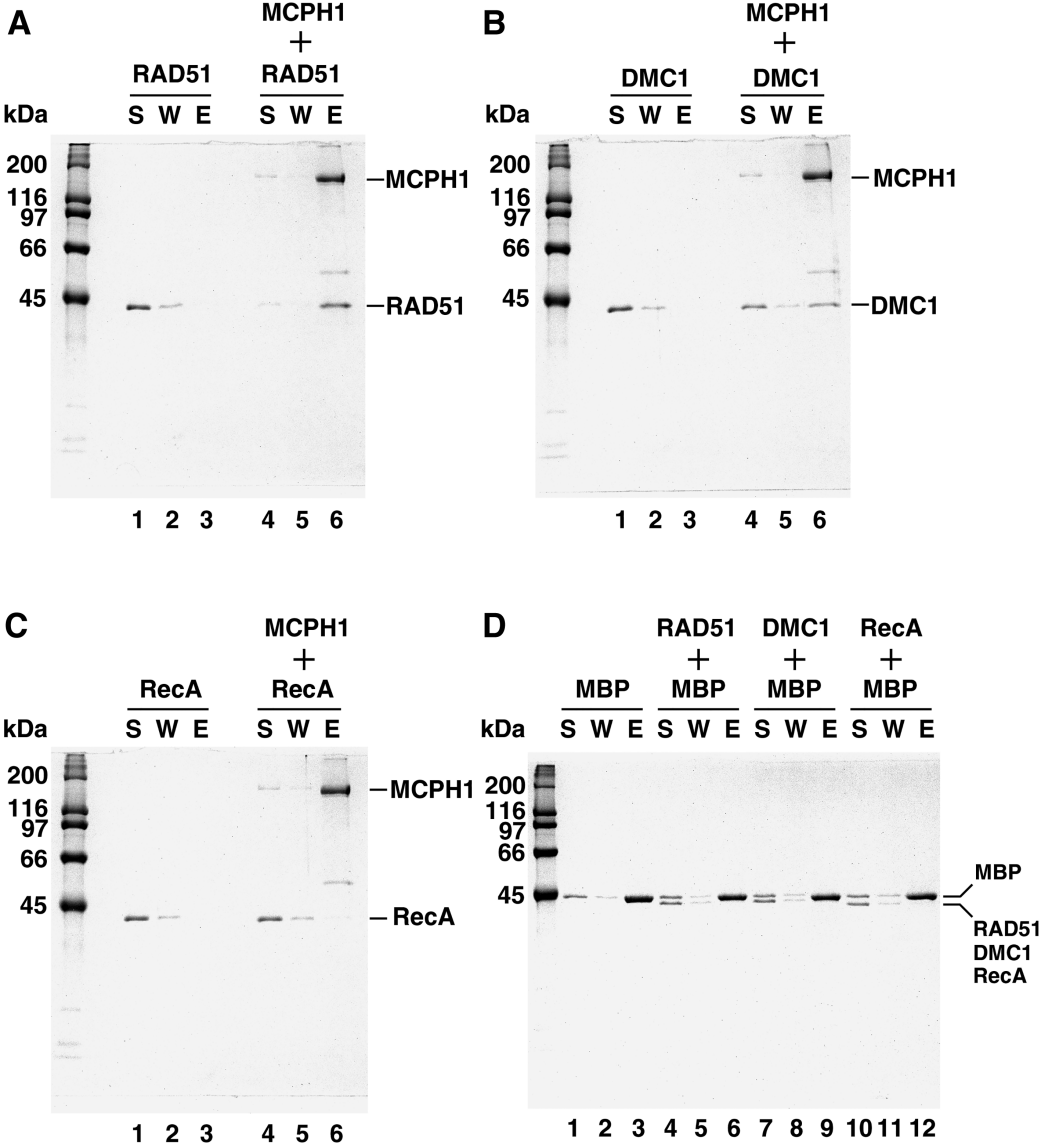
MCPH1 physically interacts with RAD51 and DMC1. In an affinity pulldown assay, purified (His)_6_-MBP-MCPH1 was pre-incubated with RAD51 (**A**), DMC1 (**B**) or RecA (**C**). The mixtures were then further incubated with Talon resin to capture protein complex via the (His)_6_-tag. The supernatant (S), wash (W), and eluate (E) from the reactions were analyzed by means of 12% SDS-denaturing polyacrylamide gels with Coomassie Blue staining. RAD51, DMC1 and RecA alone were included as controls (panels A, B and C; lanes 1–3). (**D**) Amylose resin was used to pull-down MBP proteins, with RAD51, DMC1 and RecA as negative controls.

It was reported previously that an immunocomplex of MCPH1 with BRCA2 and RAD51 represented evidence of an interaction between MCPH1 and the mitotic BRCA2–RAD51 complex (7,27). However, the inter-dependency of this potential protein-protein interaction had remained enigmatic due to lack of purified protein components for experimental analyses. Given that formation of RAD51 foci depends on MCPH1 (7,10,27), we examined the possibility of physical interaction between MCPH1 and RAD51. To do this, we conducted pulldown assays with purified proteins and found that MCPH1 directly interacts with RAD51 recombinase (Figure 3A). We further examined if DMC1 recombinase, the meiotic RAD51 homolog, can also interact with MCPH1. Notably, the meiotic recombinase DMC1 was significantly pulled down by MCPH1 (Figure 3B), expanding the potential clients of MCPH1 to meiotic regulators other than RAD51–BRCA2 complex (7). Next, we assessed if there was physical interaction between MCPH1 and the prokaryotic recombinase RecA. However, no physical interaction between MCPH1 and RecA was observed (Figure 3C). An MBP-only control was included in the pulldown experiment to rule out interaction via the MBP-tag (Figure 3D). Together, these results demonstrate direct interactions between MCPH1 and both RAD51 and DMC1. Importantly, this interaction is species-specific since no significant interaction was observed between MCPH1 and RecA.

### Multiple contacts between MCPH1 and RAD51/DMC1 recombinases

Next, we wanted to identify the MCPH1 domains responsible for the RAD51 interaction. To this end, we purified various truncated MCPH1 fragments and examined their physical interactions with RAD51 (Supplementary Figures S4A and S4B). We found that the N-terminus (amino acids 1–190, containing BRCT1), C-terminus (a.a. 628–822, containing BRCT2 and BRCT3) and the central regions (a.a. 287–383 and 384–476 containing the nuclear localization signal and a Condensin-II binding region, respectively) all contributed to interactions with RAD51 (Supplementary Figures S4C and S4D). We also sought the domains of MCPH1 responsible for interaction with DMC1 and identified similar binding domains in MCPH1 for DMC1 interaction as those for RAD51, though DMC1 interactions were weaker (Supplementary Figure S5). In summary, MCPH1 physically interacts with RAD51 and DMC1 via multiple contacts, with the BRCT domains and central region being particularly important.

### MCPH1 stabilizes RAD51–ssDNA and DMC1–ssDNA nucleoprotein filaments

**Figure 4.**
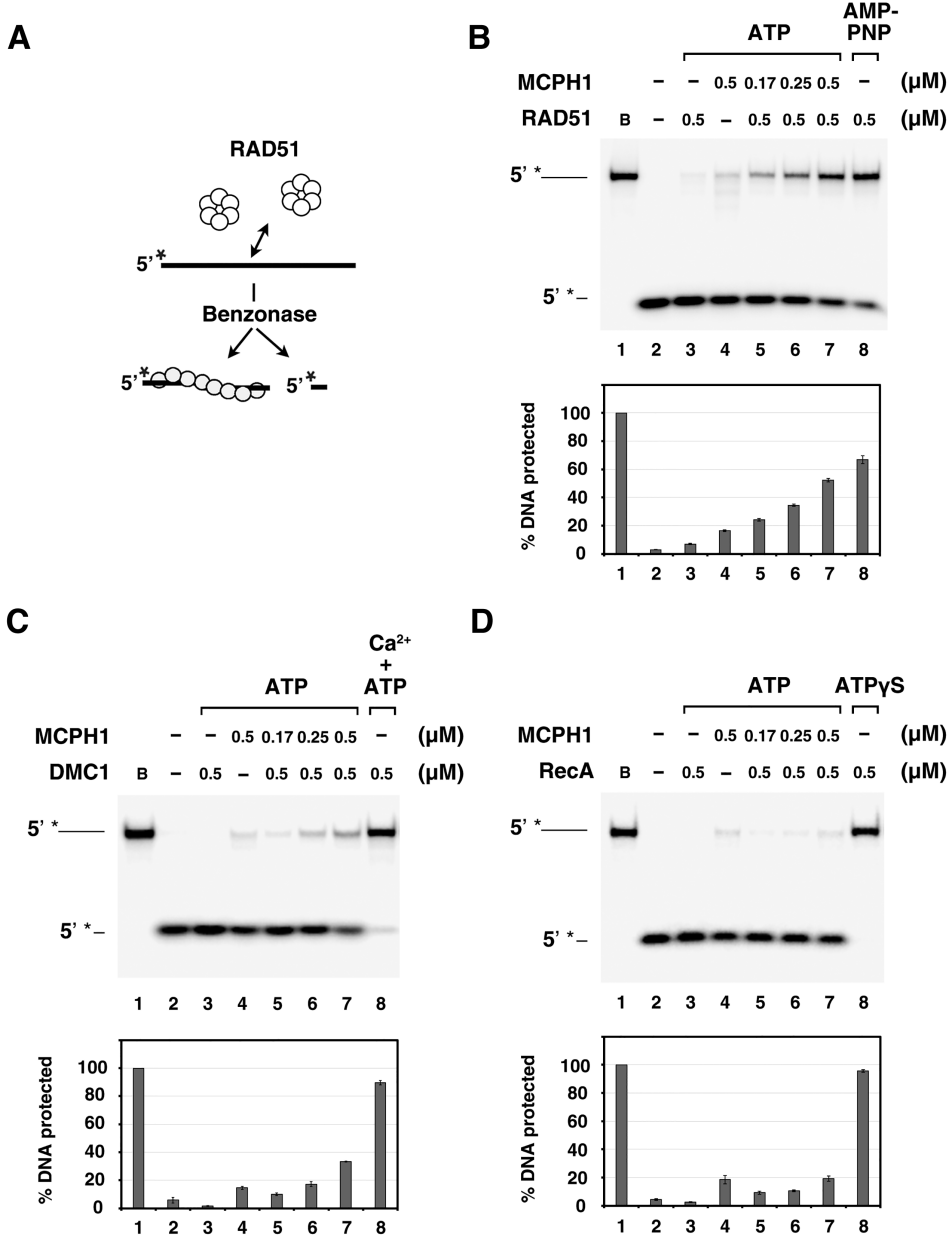
MCPH1 stabilizes RAD51 and DMC1 nucleoprotein filaments. (**A**) Schematic of our Benzonase protection assay. (**B**) The 5′-^32^P-labeled 80-mer ssDNA (1.5 μM nucleotides) was pre-incubated with RAD51 and then incubated with tag-free MCPH1 (lanes 5–7). RAD51 (lane 3) or MCPH1 alone (lane 4) were included as negative controls. RAD51 with AMP-PNP was included as a positive control. Then, Benzonase was added to challenge the filaments. The reactions were deproteinized and electrophoresed in 10% TBE polyacrylamide gels. The gels were dried and analyzed by phosphorimaging. The ^32^P-label is denoted by the asterisk. (**C**, **D**) The assay was also conducted using DMC1 (C) or RecA (D) and analyzed in the same way. DMC1 with calcium (0.5 mM) or RecA with ATPγS were included as respective positive controls. Plots of percentage DNA protected are shown below the gels. Error bars represent the standard deviation (±SD) calculated from three independent experiments.

Cell-based studies have documented that MCPH1 is required for the formation of damage-induced RAD51 foci (3,7,27). Moreover, upon depletion of MCPH1, RAD51 enrichment was absent from the chromatin fraction of cell lysate (7). Given our above-described evidence of physical interaction between MCPH1 and RAD51, we explored if MCPH1 regulates the stability of RAD51 filaments. To do so, we performed a Benzonase protection assay to monitor RAD51 filament stability. Briefly, RAD51 filaments were prepared in the presence or absence of MCPH1 and then challenged with the endonuclease Benzonase (Figure 4A). The percentage of protected DNA was used as an indicator of RAD51 filament stability. As shown in Figure 4B, susceptibility of the RAD51-associated ssDNA filaments to Benzonase was greatly attenuated by the inclusion of MCPH1 (Figure 4B). Notably, MCPH1 also slightly enhanced the stability of DMC1–ssDNA filaments (Figure 4C). MCPH1 had no effect on the stability of *E. coli* RecA (Figure 4D). Thus, MCPH1 significantly promotes the stability of RAD51- and DMC1-ssDNA filaments, and this effect is species specific.

### MCPH1 prevents RAD51- and DMC1-ssDNA filament disassembly

**Figure 5.**
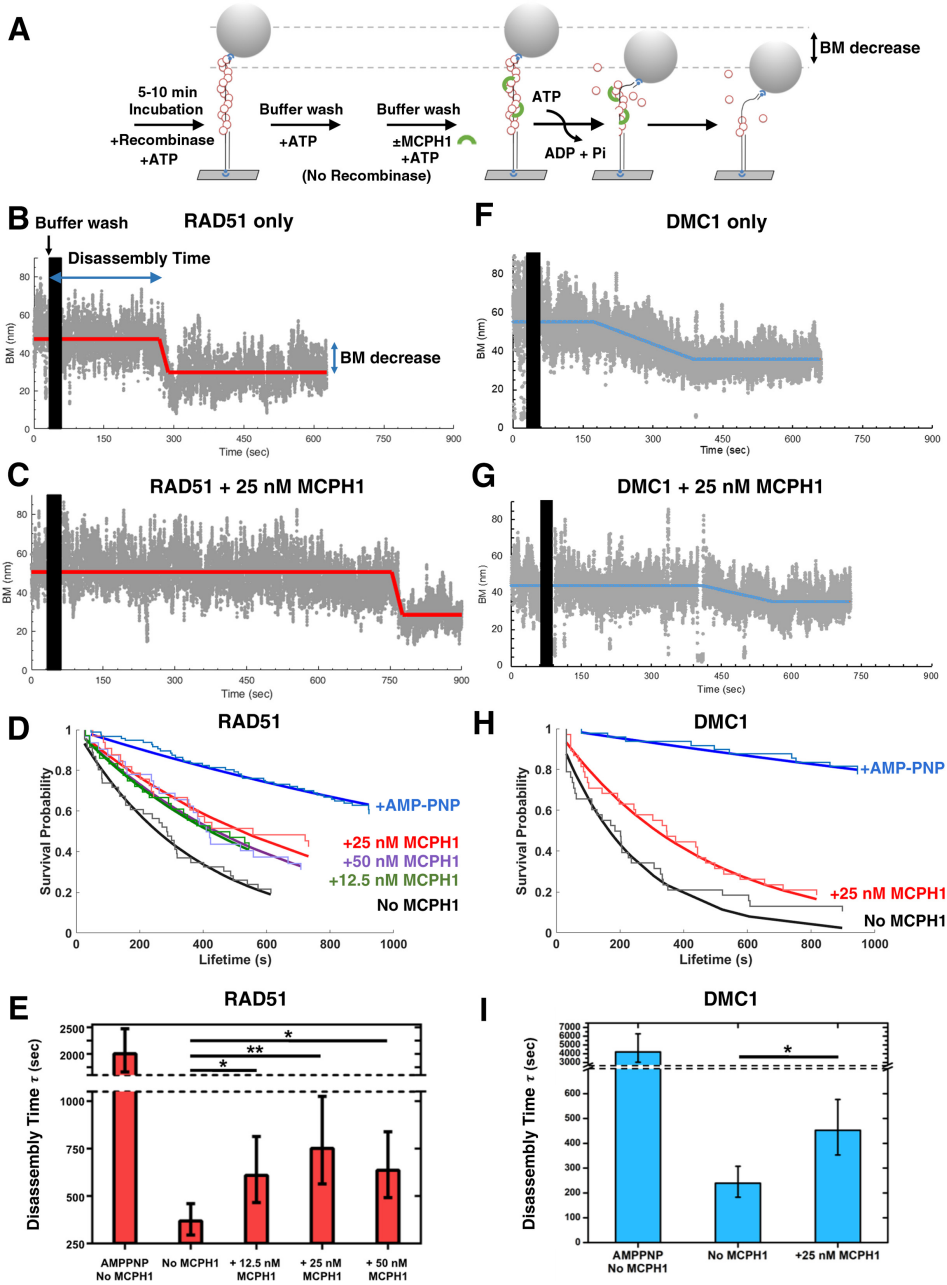
MCPH1 prevents both RAD51- and DMC1-ssDNA filament disassembly. (**A**) Schematic of our tethered particle motion disassembly experiments. The surface-bound DNA with bead was pre-incubated with RAD51-ATP or DMC1-ATP to form nucleoprotein filaments. Free recombinases were washed away and then tag-free MCPH1 was added. Disassembly of recombinase-ssDNA filaments was monitored in real-time by characterizing the change in time-lapsed Brownian motion (BM) of the DNA tethers. (**B**, **C**) Representative BM time traces of RAD51–ssDNA filament disassembly in the absence of MCPH1 (B) and in the presence of 25 nM MCPH1 (C). (**D**) Survival probability histograms of RAD51–ssDNA filament lifetime at different MCPH1 concentrations. AMP-PNP was included to replace ATP for RAD51 as a positive control. Fitted curves are based on the Kaplan-Meier estimator. (**E**) Bar graph of RAD51–ssDNA filament lifetime obtained from D. (**F**, **G**) Representative BM time traces of DMC1-ssDNA filament disassembly in the absence of MCPH1 (F) and in the presence of 25 nM MCPH1 (G). (**H**) Survival probability histograms of DMC1-ssDNA filament lifetime under different conditions. (**I**) Bar graph of DMC1-ssDNA filament lifetime compiled from (H). Data include at least 32 DNA tethers from at least three independent experiments. Error bars represent the standard deviation (±SD). Symbol meaning: *P ≤ 0.05; **P ≤ 0.01.

Parallel to our benzonase protection assay, we conducted single-molecule tethered particle motion (TPM) disassembly experiments to determine how MCPH1 affects RAD51 filaments. TPM assays monitor the Brownian motion (BM) of bead-labeled DNA tethers, enabling kinetic determination of RAD51 disassembly from DNA. RAD51-DNA filaments facilitate DNA extension and so exhibit high BM that is indicative of filament formation, as previously described (37,39). In our TPM disassembly experiments, RAD51 was first pre-incubated with surface-bound, bead-tagged gapped DNA to form RAD51 filaments that presented high BM. When excess RAD51 was removed by buffer washes, the RAD51 filaments disassemble, lowering BM magnitudes (Figure 5A). MCPH1 alone did not alter the BM of the DNA tether. The time between the last buffer wash and onset of continuous BM decline represents the time to disassemble, reflecting the stability of RAD51 filaments. As revealed by the representative time-trace shown in Figure 5B, filaments began disassembling at around the ∼250 s time-point in the presence of RAD51 alone. Remarkably, in the presence of 25 nM MCPH1, disassembly did not begin until the ∼700 s time-point (Figure 5C). Lifetime analysis of survival probability histograms (Figure 5D) reveal that the disassembly time of RAD51 filaments increased from 369 s (RAD51-only) to 751 s in the presence of 25 nM MCPH1 (Figure 5E). As a positive control, we conducted the TPM assay on RAD51 filaments formed in the presence of a non-hydrolyzable ATP analogue, AMP-PNP, which resulted in a much longer disassembly time of ∼2000 s. The extended time to disassemble of RAD51 filaments in the presence of MCPH1 was also observed at a high salt concentration (Supplementary Figure S6), a condition that enhances ssDNA binding of RAD51 (40). Moreover, MCPH1 also extended the time-point of DMC1-associated filament disassembly from ∼173 to ∼410 s in the exemplary time-traces shown (Figure 5F and G), with respective survival probability histograms (Figure 5H) revealing that the disassembly time of DMC1 filaments had increased from 239 s (DMC1 only) to 453 s in the presence of 25 nM MCPH1 (Figure 5I). Furthermore, we observed that the number of non-disassembled RAD51 or DMC1 filaments increased in the presence of MCPH1 (Supplemental Figure S7). Taken together, our Benzonase protection assay and TPM single-molecule analysis consistently demonstrate that MCPH1 stabilizes RAD51 and DMC1 nucleoprotein filaments. Notably, the enhanced effect of MCPH1 in stabilizing RAD51-associated over DMC1-associated ssDNA filaments in our TPM assays is consistent with our observations from the Benzonase protection assays (Figures 4 and 5). In an effort to further delineate the mechanism by which MCPH1 stabilizes RAD51 nucleoprotein filament, we examined ATP hydrolysis of RAD51 in the presence of MCPH1, but found that MCPH1 had no significant impact on RAD51-mediated ATP hydrolysis (Supplementary Figure S8, see also Discussion).

## DISCUSSION

In this study, we successfully established a sophisticated purification protocol for obtaining tag-free MCPH1 recombinant protein using a mammalian expression system. Our experiments reveal that MCPH1 is a *bona fide* DNA-binding protein. Importantly, our biochemical and single-molecule TPM analyses demonstrate that MCPH1 physically interacts with RAD51 and stabilizes presynaptic RAD51 filaments. Thus, we have unveiled that a MCPH family protein possesses DNA-binding ability and directly regulates recombination through a functional interaction with RAD51 recombinase.

Previous cell-based studies have revealed that depletion of MCPH1 impairs localization of BRCA2 and RAD51 at DNA break sites (7,9,10,27,28). Given that BRCA2 is a prerequisite for loading of RAD51 onto RPA-coated ssDNA, we were prompted to investigate if MCPH1 has a direct role in regulating RAD51 filament assembly. Our results document a direct species-specific interaction between MCPH1 and RAD51 protein. Importantly, our Benzonase protection assay and single-molecule TPM experiment clearly demonstrate that MCPH1 enhances the stability of presynaptic RAD51 filaments (Figures 4B and 5). It has been well documented that filament stability is regulated by ATP hydrolysis (19–22,41–45). For example, BRCA2 attenuates RAD51 ATPase activity to promote filament stability (46). Consistent with that notion, the RAD51-interacting partners SWI5-SFR1 and BCCIPβ have been shown to facilitate filament assembly by releasing ADP, which affects ATP hydrolysis of RAD51 (29,39,47). Apart from modulating ATP hydrolysis, HOP2-MND1 regulates the interaction of RAD51 with nucleotide cofactors and its DNA-binding specificity to promote DNA strand exchange (48,49). Interestingly, our data show that MCPH1 does not affect ATP hydrolysis of RAD51 (Supplementary Figure S8). Instead, MCPH1 appears to stabilize RAD51 filaments by a mechanism distinct from that deployed by BRCA2, SWI5-SFR1 and BCCIPβ to modulate RAD51 ATPase activity. We endeavored to identify MCPH1 mutant variants defective in RAD51 interaction to address the importance of protein-protein interaction for RAD51 filament stability. However, due to the complexity of the multiple contacts between MCPH1 and RAD51, we were unable to isolate a specific interaction-defective variant (Supplementary Figure S4). Thus, further studies are required to decipher how MCPH1 modulates RAD51 filament stability.

Our TPM disassembly experiment revealed that MCPH1 has a significant role in preventing RAD51 filament disassembly. Interestingly, filament disassembly was statistically similar at various MCPH1 concentrations (Figures 5D and E), indicating that substoichiometric MCPH1 is sufficient to attenuate RAD51 filament disassembly (*k*_off_). We did attempt to include even higher MCPH1 concentrations in our TPM disassembly assay to determine if MCPH1 affected the RAD51 assembly step (*k*_on_), but we were unsuccessful, likely due to MCPH1 DNA-binding activity preventing RAD51 filament assembly. On the other hand, the Benzonase protection assay probes the equilibrium state of RAD51 filaments, including both the assembly and disassembly steps. We observed increasing stability of RAD51 filaments with increasing amounts of MCPH1 upon Benzonase challenge (Figure 4B). As substoichiometric MCPH1 is sufficient to attenuate RAD51 disassembly and there was no apparent MCPH1 concentration dependency at the *k*_off_ step, the observation from Benzonase challenge that RAD51 stability is enhanced by MCPH1 in a concentration-dependent manner indicates that MCPH1 may stimulate the RAD51 assembly step (*k*_on_). That premise is consistent with findings for SWI5-SFR1 (37). Other experimental approaches should be deployed to determine how MCPH1 affects the RAD51 assembly step.

Stabilized presynaptic filaments could enhance the DNA strand exchange activity of RAD51. For example, both SWI5-SFR1 and HOP2-MND1 can stabilize RAD51 filaments and stimulate RAD51-mediated DNA strand exchange (29,37,39,48–50). We endeavored to examine if MCPH1-mediated filament stabilization contributes to the strand exchange activity of RAD51. Surprisingly, we found that MCPH1 itself exhibits a robust DNA exchange signal in D-loop assay (Supplementary Figure S9). Whether the DNA exchange activity of MCPH1 is physiologically relevant or not requires further investigation. It is worth noting that HOP2 also possesses DNA strand exchange activity (51,52), but that activity is suppressed upon association with MND1 (52). It is possible that MCPH1-mediated strand exchange activity is regulated by its client proteins. Importantly, although MCPH1 can stabilize RAD51 and DMC1 filaments, we did not observe a significant stimulatory effect of MCPH1 on their recombinase activities (Supplementary Figure S9). Thus, further studies are required to establish if filament stabilization alone is sufficient to initiate the DNA strand exchange process. Intriguingly, Ca^2+^ has been shown to stabilize fission yeast Dmc1 filaments but does not enhance strand exchange activity (53).

In addition to functional interaction with RAD51, we also reveal that MCPH1 physically interacts with DMC1 and promotes DMC1 filament stability, albeit to a lesser extent than for RAD51. Consistent with this finding, *Mcph1*^−/−^ mice exhibit a sterile phenotype and *Mcph1*-deficient spermatocytes are arrested prior to the pachytene stage and display aberrant chromosomal synapsis (7). Consequently, we speculate that MCPH1 plays an important function in regulating both RAD51 and DMC1 during meiosis. The importance of MCPH1-DMC1-RAD51 interaction for meiotic recombination requires further investigation.

Cell-based studies have documented that MCPH1 depletion impairs the formation of both BRCA2 and RAD51 foci. Furthermore, protein truncation analysis has revealed that the C-terminal BRCT domain of MCPH1 is a prerequisite for BRCA2 foci formation (27). Interestingly, our *in vitro* affinity pulldown assays show that both the N-terminal and C-terminal BRCT domains contribute to the physical interaction of MCPH1 with RAD51 (Supplementary Figure S4), indicating that MCPH1 may act as a scaffold to bind both BRCA2 and RAD51 through multiple epitopes. It is worth noting that MCPH1 acts upstream of the BRCA2–RAD51 axis based on the observation that damage-induced MCPH1 foci are normal in *Brca2*-deficient cells (27). Consistent with that notion, it has been reported previously that MCPH1 acts early in the DNA damage response as a DNA damage sensor through its interaction with γ-H2AX (14–18). Based on all of these findings, we hypothesize that MCPH1 targets to DSB sites via a link with γ-H2AX upon a DNA damage response and that MCPH1 then recruits both BRCA2 and RAD51 to break sites. Importantly, our biochemical and single-molecule TPM studies have demonstrated that MCPH1 also possesses an additional function in stabilizing RAD51 presynaptic filaments. Further studies are necessary to establish the functional significance of the MCPH1–RAD51–BRCA2 complex. The protein purification protocol and MCPH1 characteristics we document herein should greatly facilitate future efforts directed at delineating the functional relationship between BRCA2 and MCPH1 that is responsible for regulating RAD51 filament assembly.

## Supplementary Material

gkaa636_Supplemental_File
