# Supplementary material for: Microcephaly family protein MCPH1 stabilizes RAD51 filaments
Source: Nucleic Acids Res. 2020 Jul 31;48(16):9135–46. doi: 10.1093/nar/gkaa636 (PMC7498314; doi:10.1093/nar/gkaa636)
Supplement: gkaa636_Supplemental_File [file gkaa636_supplemental_file.pdf]

Microcephaly family protein MCPH1 stabilizes RAD51 filaments

Hao-Yen Chang[1], Chia-Yi Lee[1], Chih-Hao Lu[2], Wei Lee[2], Han-Lin Yang[2], Hsin-Yi Yeh[1], Hung-Wen Li [2,*], and Peter Chi[1,3,*]

[1] Institute of Biochemical Sciences, National Taiwan University, No. 1, Sec. 4, Roosevelt Road, Taipei, 10617 Taiwan
[2] Department of Chemistry, National Taiwan University, No. 1, Sec. 4, Roosevelt Road, Taipei, 10617 Taiwan
[3] Institute of Biological Chemistry, Academia Sinica, 128 Academia Road, Section 2, Nankang, Taipei 11529, Taiwan

* To whom correspondence should be addressed.
Peter (Hung Yuan) Chi
Address:
Institute of Biochemical Sciences
College of Life Science
National Taiwan University
No. 1, Sec. 4, Roosevelt Road, Taipei, 10617 Taiwan
Email: peterhchi@ntu.edu.tw
Phone: 886-2-23665573
Fax: 886-2-23635038

Hung-Wen Li
Address:
Department of Chemistry
College of Science
National Taiwan University
No. 1, Sec. 4, Roosevelt Road, Taipei, 10617 Taiwan
Email: hwli@ntu.edu.tw
Phone: 886-2-3366-4089
Fax: 886-2-3366-8671

Running Title: *Biochemical significance of MCPH1 in RAD51-mediated homologous recombination*

SUPPLEMENTARY DATA

SUPPLEMENTARY MATERIAL AND METHODS

Gel-filtration analysis

Tag-free MCPH1 (85 μg) was diluted in 0.5 ml buffer A with 300 mM KCl and analyzed by gel-filtration chromatography through a Superose increase 6 10/300 GL column (GE Healthcare). The following protein size markers were included: Blue Dextran 2000 (2000 kDa), Thyroglobulin (669 kDa), Ferritin (440 kDa), Aldolase (158 kDa), and Conalbumin (75 kDa). The reaction was conducted in a 1-column volume (24 ml) of buffer A with 300 mM KCl and fractionated out as 1 ml per tube. The eluted fractions were analyzed by Western blotting.

ATP hydrolysis assay

$(His)_6$-tagged mouse RAD51 (0.5 μM) was pre-incubated to form nucleoprotein filaments in reaction buffer C containing 2 mM $MgCl_2$ and 0.2 mM ATP with or without 80-mer Oligo 1 ssDNA (1.5 μM nucleotides) at 37 °C for 5 min. Then, tag-free MCPH1 (0.17 μM) protein was added into the reaction for a further 5 min. After incubation, γ-^{32}P-ATP (1.8 μCi) was added as an indicator of hydrolysis and to initiate the reaction. Aliquots (2 μl) were taken at the indicated time-points and mixed with an equal volume of 500 mM EDTA to stop the reaction. ATP hydrolysis was determined by thin-layer chromatography on a polyethyleneimine sheet (Merck) using 0.15 M LiCl and 0.5 M formic acid as the developing buffer. The chromatography sheet was air-dried and subjected to phosphorimaging analysis. The percentage of ATP hydrolysis was calculated from the ratio of the intensity of hydrolyzed γ-^{32}Pi phosphate to total γ-^{32}P (including free γ-^{32}Pi and γ-^{32}P-ATP).

D-loop assay

^{32}P-labeled 90-mer Oligo 11 (2.4 μM nucleotides) was incubated with indicated amounts of $(His)_6$-tagged mouse RAD51 or $(His)_6$-tagged mouse DMC1 in 10.5 μl of buffer C with 0.1 mM ATP and 2 mM $MgCl_2$ for 5 min at 37 °C. Then, the indicated amount of tag-free MCPH1 was added into the reaction for a further 5 min. The reaction was initiated by adding pBluescript replicative form I DNA (190 μM base

pairs) to a 12.5 µl final volume. After 5 min of incubation, a 5 µl aliquot was removed and the reaction was terminated by addition of a final concentration of 0.05% SDS and proteinase K (0.8 mg/ml) and incubation at 37 °C for 15 min. The samples were analyzed on 0.9% agarose gels in TBE buffer at 4 °C. Gels were dried onto DE81 paper and subjected to phosphorimaging analysis. The percentage of product formation was obtained from the ratio of paired DNA signal intensity to that of total DNA species, with normalization to DNA blank as 0%.

SUPPLEMENTARY FIGURES

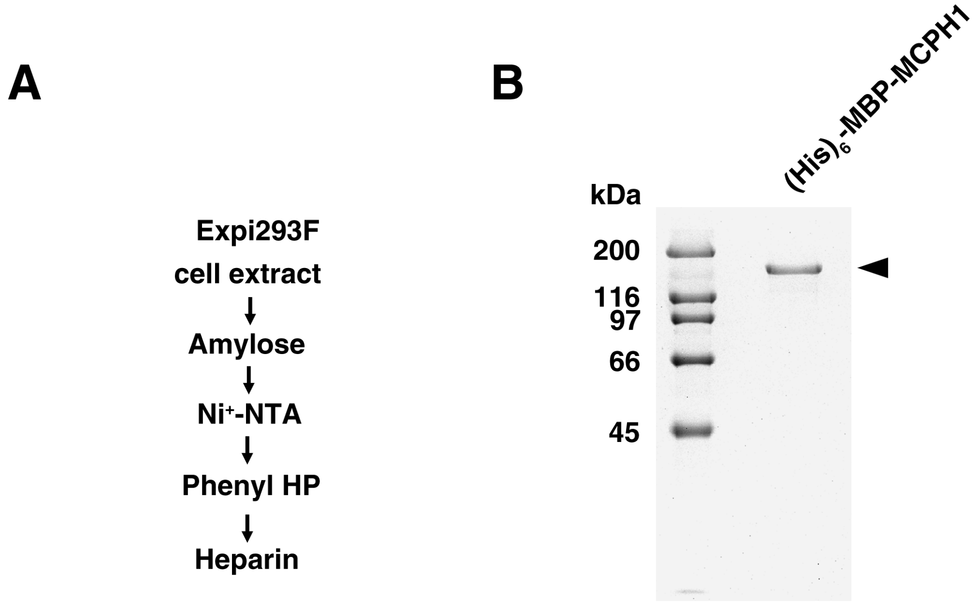

Supplementary Figure 1. Purification of $(His)_6$-MBP-tagged MCPH1 protein

(A) Schematic of the purification protocol. The cell extract was fractionated through Amylose, Ni^{+}-NTA, Phenyl HP, and Heparin columns. **(B)** Purified $(His)_6$-MBP-tagged MCPH1 (1 µg) was analyzed in a 12% SDS-denaturing polyacrylamide gel with Coomassie Blue staining.

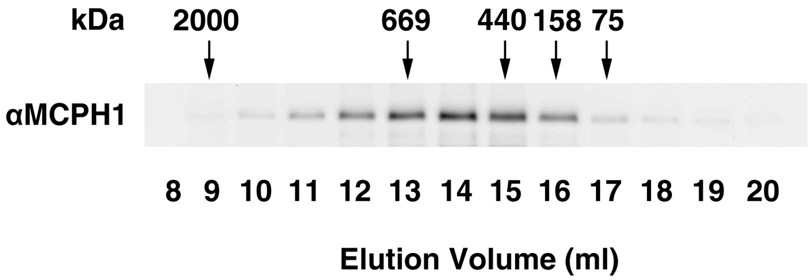

Supplementary Figure 2. MCPH1 exhibits soluble oligomers in solution

Tag-free MCPH1 was analyzed in a Superose 6 increase 10/300 GL column. The eluted fractions were analyzed by Western blot with anti-MCPH1 antibody. The corresponding molecular weight was determined from comparison to the molecular markers Blue Dextran 2000 (2000 kDa), Thyroglobulin (669 kDa), Ferritin (440 kDa), Aldolase (158 kDa), and Conalbumin (75 kDa).

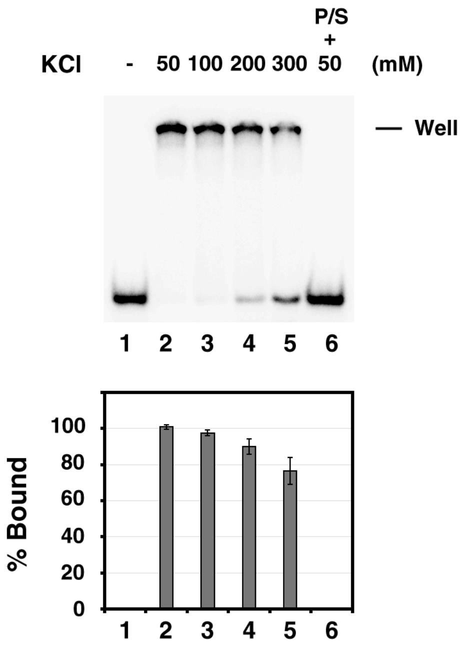

Supplementary Figure 3. MCPH1-ssDNA complex resists salt challenge

Tag-free MCPH1 (250 nM) and ssDNA (Oligo 5, 750 nM nucleotides) were incubated in the presence of the indicated KCl concentrations. In lane 6, the reaction mixture was treated with proteinase K and SDS (P/S) to release the DNA from the nucleoprotein complex. Percentage bound DNA is shown below the gel. Error bars represent standard deviation (±SD) from three independent repeats.

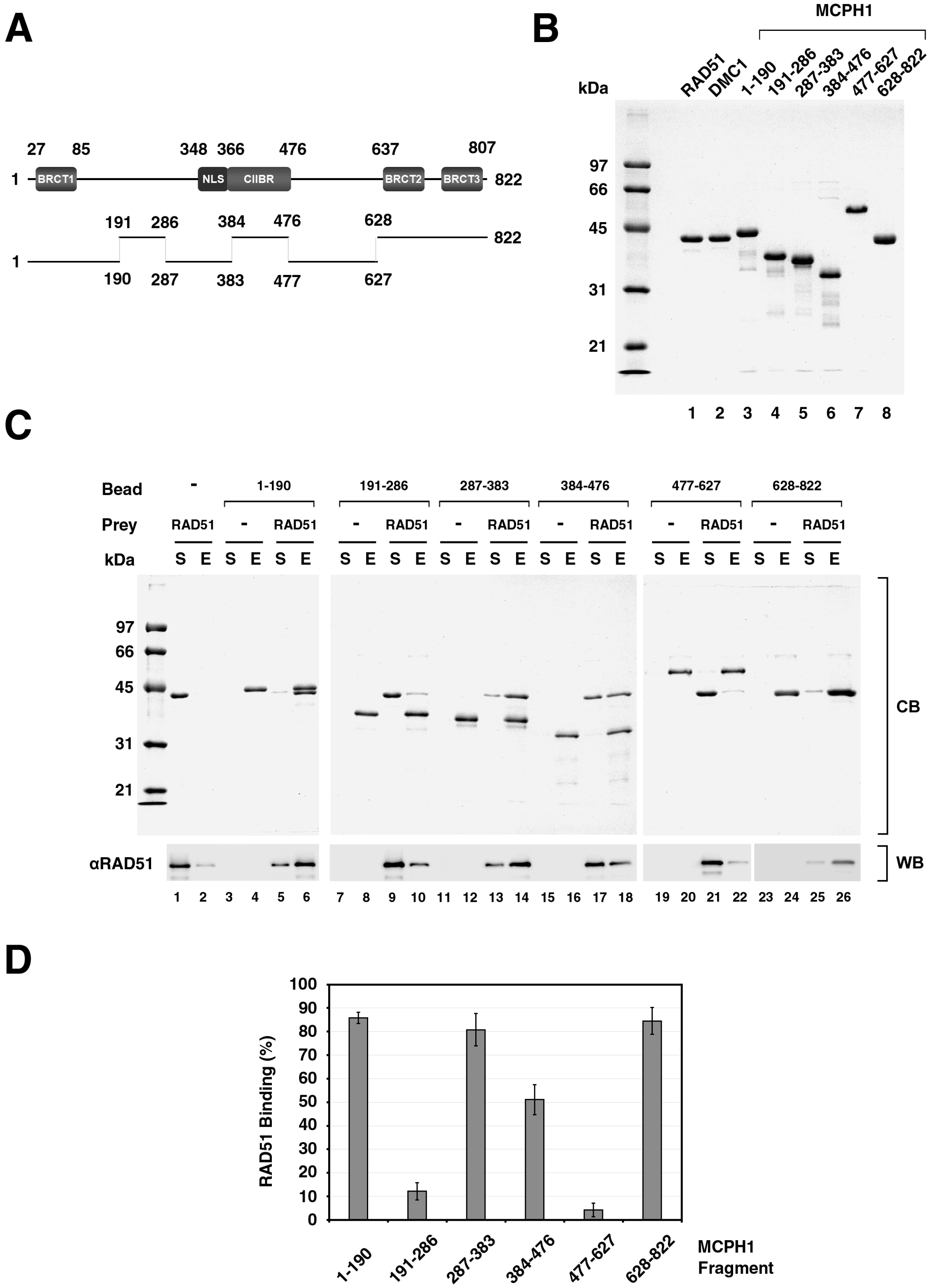

Supplementary Figure 4. MCPH1 interacts with RAD51 via multiple contacts

(A) Schematic of MCPH1 truncation variants**. (B)** Purified Strep-tagged RAD51, Strep-tagged DMC1, and $(His)_6$-SUMO-tagged MCPH1 fragments (1 µg each) were analyzed in a 12% SDS-denaturing polyacrylamide gel with Coomassie Blue staining.

(C) $(His)_6$-tagged MCPH1 fragments were incubated with RAD51, and the mixtures were further incubated with TALON resin to capture protein complex via the $(His)_6$-tag. The supernatant (S) and eluate (E) from pull-down reactions were analyzed by 12% SDS-denaturing polyacrylamide gel with Coomassie Blue staining (CB), with Western blotting (WB) using RAD51 antibody included for comparison. **(D)** Quantification of the percentage of RAD51 in eluted fractions from **C**. Error bars represent the standard deviation (±SD) from duplicate independent repeats. Abbreviations: BRCT, BRCA1 C-terminal; NLS, nuclear localization signal; CIIBR, Condensin II binding region.

A

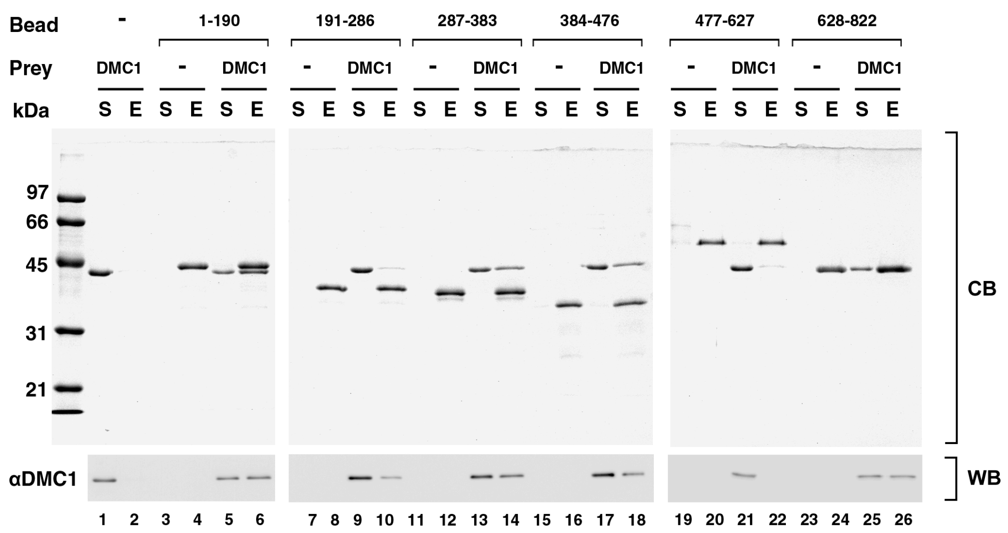

B

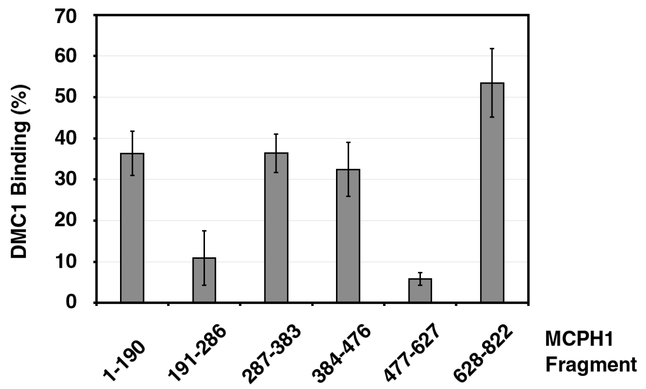

Supplementary Figure 5. MCPH1 interacts with DMC1 via multiple contacts

The experiment was conducted as described in Supplementary Figure 4, except that RAD51 was replaced with DMC1. **(A)** The supernatant (S) and eluate (E) from pull-down reactions were analyzed by 12% SDS-denaturing polyacrylamide gel with Coomassie Blue staining (CB), with Western blotting (WB) using DMC1 antibody included for comparison. **(B)** Quantification of the percentage of DMC1 in eluted fractions from **A**. Error bars represent the standard deviation (±SD) from duplicate independent repeats.

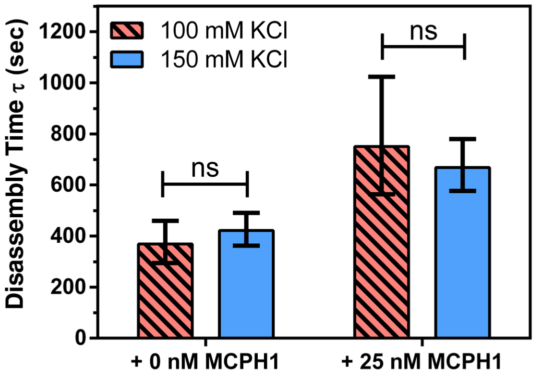

Supplementary Figure 6. MCPH1 prevents RAD51-ssDNA filament disassembly in high salt conditions

The tethered particle motion disassembly experiment was conducted as described in Figure 5. Potassium concentration was increased from regular (100 mM KCl) to high salt (150 mM KCl) conditions as indicated to prevent binding of RAD51 onto the dsDNA handle. RAD51 only (0 nM MCPH1, left bars) was included for comparison. Error bars represent the standard deviation (±SD) from at least three independent experiments. Symbol meaning: ns, not significant, $P > 0.05$.

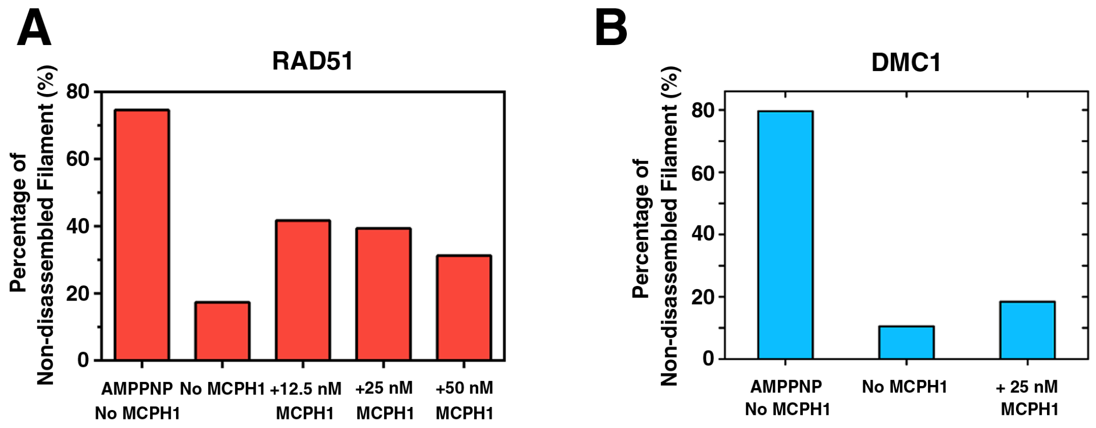

Supplementary Figure 7. MCPH1 increases the proportion of non-disassembled RAD51-ssDNA and DMC1-ssDNA filaments

Total percentage of non-disassembled filaments of RAD51 **(A)** and DMC1 **(B)** was calculated from data shown in Figure 5. RAD51 or DMC1 with AMP-PNP was included as a positive control, and MCPH1-free experiments were included as a negative control. Amounts of tag-free MCPH1 added to the reactions are shown. Inclusion of MCPH1 results in greater survival of RAD51-ssDNA and DMC1-ssDNA filaments. Percentages were calculated by compiling all DNA tethers from at least three independent experiments.

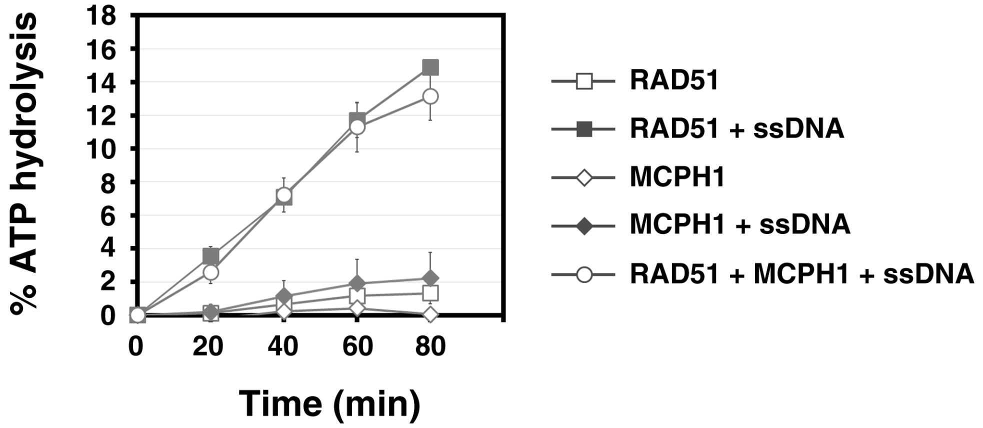

Supplementary Figure 8. RAD51 ATPase activity is not altered by MCPH1

RAD51 was pre-incubated alone (RAD51) or with 80-mer single-strand DNA (RAD51+ssDNA), and further incubated with tag-free MCPH1 (RAD51+MCPH1+ssDNA). MCPH1 alone (MCPH1) or with single-strand DNA (MCPH1+ssDNA) were included as controls. After pre-incubation, γ-^{32}P-ATP was added as an indicator of hydrolysis for the indicated times. The error bars represent the standard deviation (±SD) calculated from three independent experiments.

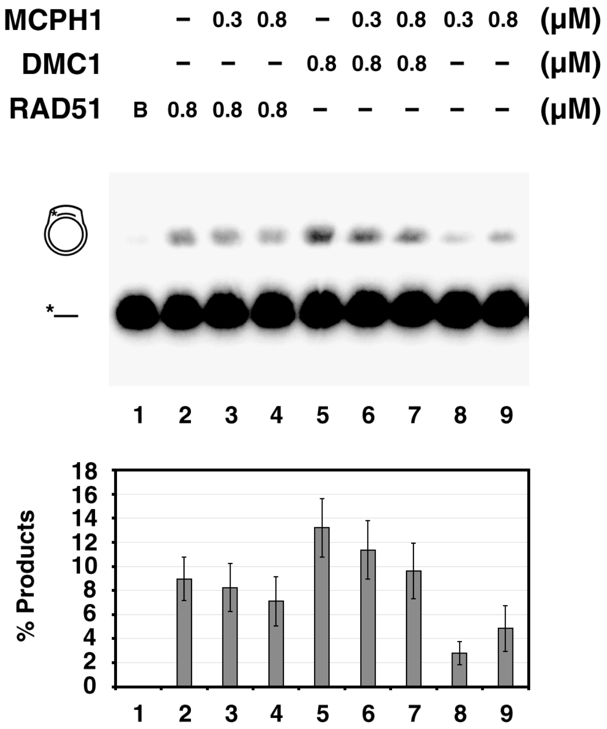

Supplementary Figure 9. D-loop formation of RAD51 or DMC1 in the presence of MCPH1

The D-loop assay was conducted as described in Supplementary Material and Methods. RAD51 (Lane 2), DMC1 (Lane 5), and MCPH1 alone (Lanes 8 and 9) were included for comparison. Addition of MCPH1 did not appear to enhance RAD51- or DMC1-mediated D-loop formation. Notably, MCPH1 alone exhibits significant D-loop activity. Percentage product formed is shown below the gel. Error bars represent the standard deviation (±SD) from three independent repeats.

Supplementary Table 1. Oligo sequences used in this study

Oligo	Sequence (5’ to 3’)
Oligo 1	TTATGTTCATTTTTTATATCCTTTACTTTATTTTCTCTGTTTATTCATTTA<br>CTTATTTTGTATTATCCTTATCTTATTTA
Oligo 2	GCCAGGGACGGGGTGAACCTGCAGGTGGGCGGCTGCTCATCGTAG<br>GTTAGTATCGACCTATTGGTAGAATTCGGCAGCGTCATGCGACGGC
Oligo 3	GCCGTCGCATGACGCTGCCGAATTCTACCACGCTACTAGGGTGCCT<br>TGCTAGGACATCTTTGCCCACCTGCAGGTTCACCCCGTCCCTGGC
Oligo 4	AAGATGTCCTAGCAAGGCACCCTAGTAGC
Oligo 5	ACGCTGCCGAATTCTACCAGTGCCTTGCTAGGACATCTTTGCCCACC<br>TGCAGGTTCACCC
Oligo 6	GGGTGAACCTGCAGGTGGGCAAAGATGTCCATCTGTTGTAATCGTCA<br>AGCTTTATGCCGT
Oligo 7	ACGGCATAAAGCTTGACGATTACAACAGATCATGGAGCTGTCTAGAG<br>GATCCGACTATCG
Oligo 8	CGATAGTCGGATCCTCTAGACAGCTCCATGTAGCAAGGCACTGGTAG<br>AATTCGGCAGCGT
Oligo 9	GGGTGAACCTGCAGGTGGGCAAAGATGTCCTAGCAAGGCACTGGTA<br>GAATTCGGCAGCGT
Oligo 10	AGGCACTGGTAGAATTCGGCAGCGT
Oligo 11	AAATCAATCTAAAGTATATATGAGTAAACTTGGTCTGACAGTTACCAA<br>TGCTTAATCAGTGAGGCACCTATCTCAGCGATCTGTCTATTT

Supplementary Table 2. Oligos used in this study

Labeled (5’-^{32}P)	Substrate	Annealed with	Illustration (Asterisk: P^{32}-labeled)
Oligo 3	ssDNA	-	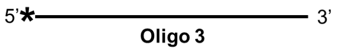
	D-loop	Oligo 2, Oligo 4	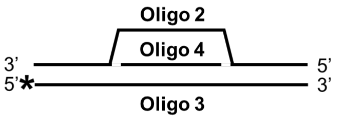
Oligo 5	ssDNA	-	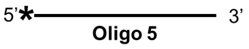
	dsDNA	Oligo 9	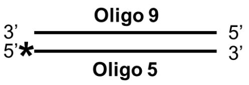
	3’-overhang	Oligo 10	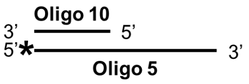
	Holliday junction	Oligo 6, Oligo 7, Oligo 8	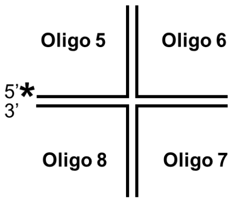
